# The Influence of Health, Marriage and Cohabitation on Physical Inactivity: Evidence from SHARE for 12 European Countries

**DOI:** 10.21203/rs.3.rs-10795133/v1

**Published:** 2026-08-25

**Authors:** Hans Gevers

**Affiliations:** Estonian Business School

**Keywords:** physical inactivity, marriage, cohabitation, partnership, health, depression, Europe, SHARE, longitudinal

## Abstract

Physical inactivity is a potential cost driver for health insurance as it may trigger undesirable health effects. In this study, the influence of health, marriage and cohabitation on physical inactivity is explored by means of fixed-effects logistic panel, random-effects logistic panel, and pooled logistic estimators. All three rely on data from the Survey of Health, Ageing and Retirement in Europe (SHARE) of 42,379 respondents aged 50 to 89 years old in 2015 from 12 European countries for the period 2015 to 2022. The analysis supports the positive influence of education and partnership on physical inactivity, as well as the negative one of ageing, illness, unemployment, limitations, and depression. In particular for partnership, the potentially positive influence on physical inactivity of being back single when higher depressed is surprising. Additionally, employed respondents relate to higher odds of reporting physical inactivity relative to retired respondents, suggesting that physical labour may be limited in their jobs. Lastly, Swedes, Danes, and Swiss relate to the lowest physical inactivity levels, and Italians to the highest. In conclusion, this study supports the importance of health and partnership for physical inactivity.

## Introduction

Physical inactivity is commonly known to relate to unhealthy conditions, like obesity (Hilz & Wagner, 2018). Despite that marriage and cohabitation may as well trigger being overweight, both are primarily expected to positively influence health and wealth (Averett et al., 2013; Pollard & Harris, 2013). Stimpson, Wilson, and Peek (2012) also document this positive influence and conclude that poverty and non-marriage jointly result in more physical inactivity. This study econometrically explains physical inactivity by health, wealth, and relationship status. This tripartite suggests applying the Social Cognitive Theory (CST) in the context of health management. Fitting the theory implies that wealth and health are personal factors, relationship status is an external environment factor, and physical inactivity a behavioural factor. In CST, all operate as “interacting determinants that influence each other bidirectionally”. Illustrating this triadic reciprocal causation goes beyond the aim of this study. However, this study acknowledges that physical inactivity, as unhealthy behaviour, depends on self-efficacy, i.e. a CST-cornerstone or “people’s beliefs that they can exert control over their own motivation, thought processes, emotional states, and patterns of behaviour”. It highlights the need for the physically inactive to be able and motivated to change their potentially unhealthy behaviour while being exposed to social pressures, situational constraints, and the like (Bandura, 1994; Wood & Bandura, 1989). In this study, the determinants are identified, yet not their bidirectionality or causality. The subsequent sections cover the literature review, data, methodology, results, discussion, and conclusion.

### Literature Review

The existing literature identifies a variety of relevant findings related to physical inactivity. The latter is expected to come with a societal cost primarily arising from increased health risks. E.g., inactive Estonians are estimated to cost the public health insurance millions of euros annually. A cost that can, according to Saavaste, Baburin, Leinsalu, and Reile (2025), be minimised through prevention. Duijvestijn, de Wit, van Gils, and Wendel-Vos (2023) confirm in their systematic review the expectation that physical inactivity triggers higher healthcare costs. However, they also expect that an increased physically active population may, in the long term, result in higher healthcare costs (e.g., due to increased sports injuries). To some extent, these costs may be regarded as desirable. Li and Sun (2022) illustrate for China that physically inactive adults report 24 per cent lower odds of seeking healthcare relative to their active counterparts. They consider it primarily relevant for the early detection and treatment of diseases. Zhao et al. (2022) consider that governmental interventions ought to target the unhealthy lifestyle, in particular for those who report both inactivity and being overweight, and subsequently face higher personal out-of-pocket health expenditures and impose an additional burden on the healthcare system. Overall, overcoming the negative consequences of physical activity is not self-evident, given that the ideal healthy and healthcare-lean lifestyle is hard to define.

Beyond the financial cost, physical inactivity relates to a physical and mental cost. E.g., for the former, physical inactivity directly influences the development of chronic pain, as illustrated by Lindell and Grimby-Ekman (2022) based on a Swedish sample. The influence may be more explicit in interaction with bad sleep and stress. For the latter, physical inactivity primarily relates to depression. E.g., Nygård et al. (2025) explore physical activity among Swedes aged 65 years and older with a logistic estimator integrating gender, age, country, education, and the like. They find physical inactivity to relate for some to higher levels of psychological distress. This finding is supported by Asp, Simonsson, and Molarius (2025) who conclude that Swedish older people more often report symptoms of anxiety and self-reported depression when physically inactive. Also, Zheng, He, Xu, Zheng, and Gu (2022) consider physical inactivity strengthening depression. The influence may even hold in two directions, as concluded by Zhao et al. (2023) who conclude that a two-way causal relationship exists between physical inactivity and major depressive disorders. Evidently, besides health, a variety of other factors may explain physical inactivity. Pengpid and Peltzer (2022) find that Indian older adults are more likely to report inactivity when reporting a higher socioeconomic status, higher states of depression and/or worse health. In contrast, they consider being married, higher educated and socially active to negatively relate to physical inactivity. Primarily, the influence of marriage is of interest in this study as it is expected to come with several positive influences and benefits. E.g., Gevers (2026b) highlights the positive influence of partnership on financial distress. Stimpson et al. (2012) find that marriage results in less inactivity, in particular for those in poor health. On top, they consider inactivity determined by the extent to which resources are shared through marriage, even in a more explicit way than through cohabitation. Similarly, Hilz and Wagner (2018) consider inactive behaviour to be tempered by marriage, yet acknowledge that it may just as well trigger being overweight. The benefits of partnership also apply to Japan, where singles are more likely to be physically inactive. The latter also holds for non-working respondents. Interestingly, the study of Sumimoto et al. (2021) documents no significant effects of educational attainment on physical inactivity. In conclusion, the existing literature supports the broad expectation that health, partnership, and wealth influence physical inactivity. This expectation is the underpinning of this study for the econometric analysis completed by means of a large dataset. The latter is specified in the subsequent section.

### Data

This study uses data from SHARE, which is a project of the European Research Infrastructure Consortium (ERIC) (Bergmann et al., 2019; Börsch-Supan et al., 2013). It realises a recurring health survey focused on the effects of health, social, economic, and environmental policies. It provides information on up to 42,379 respondents from the SHARE Waves 6 to 9 (SHARE-ERIC, 2024a, 2024b, 2024c, 2024d). The respondents are aged 50 to 89 years old in 2015, and are carried forward to 2022. Table 1 on the following page summarises the variables in the sample.

The sample consists of 17 variables of which two cover the survey years, 2015, 2017, 2020, and 2022, as well as the unique identification number of each respondent. Both variables (not listed in Table 1) are preferably used as levels for the panel estimators. The remaining 15 variables are listed in Table 1. Physical inactivity is the binary dependent variable with 0 No and 1 Yes. It originally covered a category Not applicable, which has been dropped (i.e. 24,644 observations). Of the 14 independent variables, four variables are considered continuous, namely Age, Years of education, Body mass index, and Depression. A respondent is between 50 and 96 years old during the observed period, and 70 years old on average. The same respondent received 11 years of education on average, while a maximum of 30 years is observed in the sample. Overweight appears to thrive among the respondents as the average Body mass index is 27. Also underweight as well as obesity occur in the sample given, respectively, the minimum of 13 and the maximum of 99. The last continuous variable is Depression, of which its 13 categories are assumed to be on a continuous scale. The remaining 10 independent variables are used as categorical variables. Gender reports 1 Male and 2 Female. Total household income is winsorized at the 99th percentile, and subsequently converted into a categorical variable with 0 No income, 1 Below country average income, and 2 Above country average income. Job situation consists of six categories, i.e. 0 Retired, 1 Employed or self-employed, 2 Unemployed, 3 Permanently sick, 4 Homemaker, and 5 Other (e.g., student or volunteer). It originally included a category Not applicable, which has been dropped (i.e. 1,089 observations). Besides the continuous health indicators Body mass index and Depression, two categoricals additionally reflect health and may be included in the econometric analysis separately. As such, the analysis integrates either the Self-perceived health status with five categories from 1 Excellent to 5 Poor, or the binary Limitation in activities with 0 Not limited or 1 Limited. A last but one categorical variable is, like the survey years, time-invariant and represents the respondent’s country of citizenship. A total of 12 European countries are covered by this variable, being Austria, Germany, Sweden, Spain, Italy, France, Denmark, Greece, Switzerland, Belgium, Czech Republic, and Poland (the original SHARE-number is referred to in Table 1). The last variables reflect relational features and are constructed as illustrated by Gevers (2026b). A first categorical is labelled Marital status and holds four categories, namely 0 Officially with partner, 1 Away from partner, 2 Never married and 3 Lost partner. The latter two are original as provided by SHARE. The former is a combination of the original SHARE-categories Married, living with spouse and Registered partnership. The category Away from partner is a combination of the original categories Married, not living with spouse and Divorced. A second relational feature is the year-on-year change in the Marital status and is documented by the variable Change in marital status, which indicates the status change relative to the previous survey year. The marital status is assumed to be unchanged in the first survey year. The variable aims to capture whether a respondent is married or not in a following survey year. The variable allows a respondent to enter and exit a marriage multiple times across the time horizon. The difference between the marriage exit and entry is of interest, implying that the variable is not interpreted relative to its (meaningless) baseline. Three categories are reported by the variable Change in marital status, i.e. 0 No longer officially together, 1 All other situations, and 2 Newly officially together. The Relationship variables are constructed in the same way as the Marital variables. However, Relationship status covers merely two categories, i.e. 0 Not single or 1 Single, and its change is covered with 0 Hooked up, 1 Unchanged, and 2 Back single. Both status, Marital as well as Relationship, reflect the same concept as supported by their strong Spearman correlation of 0.855 (see Table 4 in Appendix). As Marital status is assumed to cover the influence of marriage, and Relationship status the influence of cohabitation, both are used in the analysis, yet in separate equations. Similarly, the Spearman correlation of Limited in activities and Self-perceived health status of 0.541 is considered too high given that both variables express similar concepts. As such, also these two variables are not jointly regressed. The high Spearman correlation between Age and Job situation of −0.477 is tolerated given that it remains under a 0.600 threshold and regards different concepts and variable types.

To provide additional insight into the sample, four figures are subsequently plotted to document the primary variables of interest in this study, namely the physical inactivity, relationship status, marital status, and self-perceived health status.

**Figure 1:**
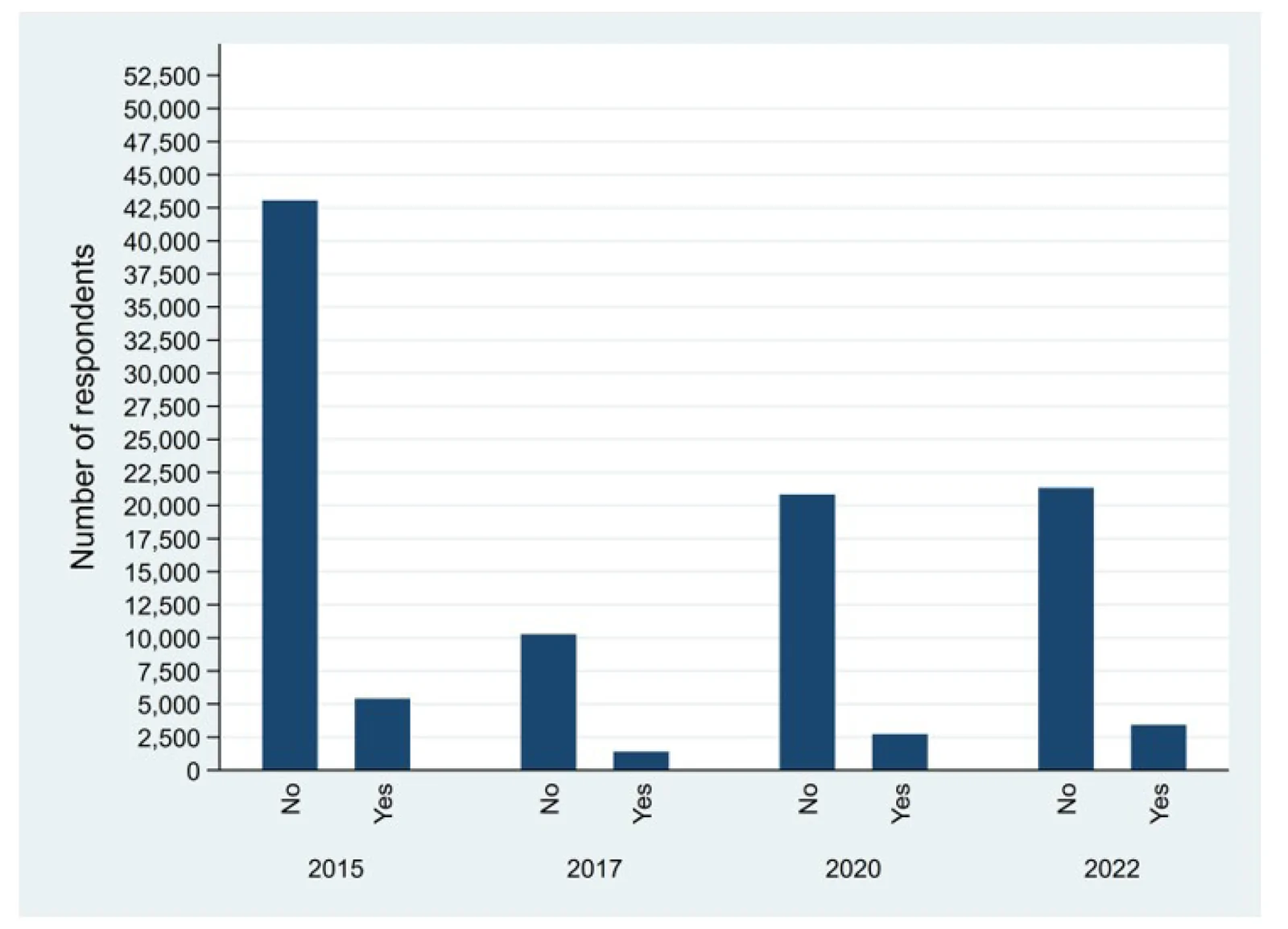
Number of Respondents for Physical inactivity and per Survey year

**Figure 2:**
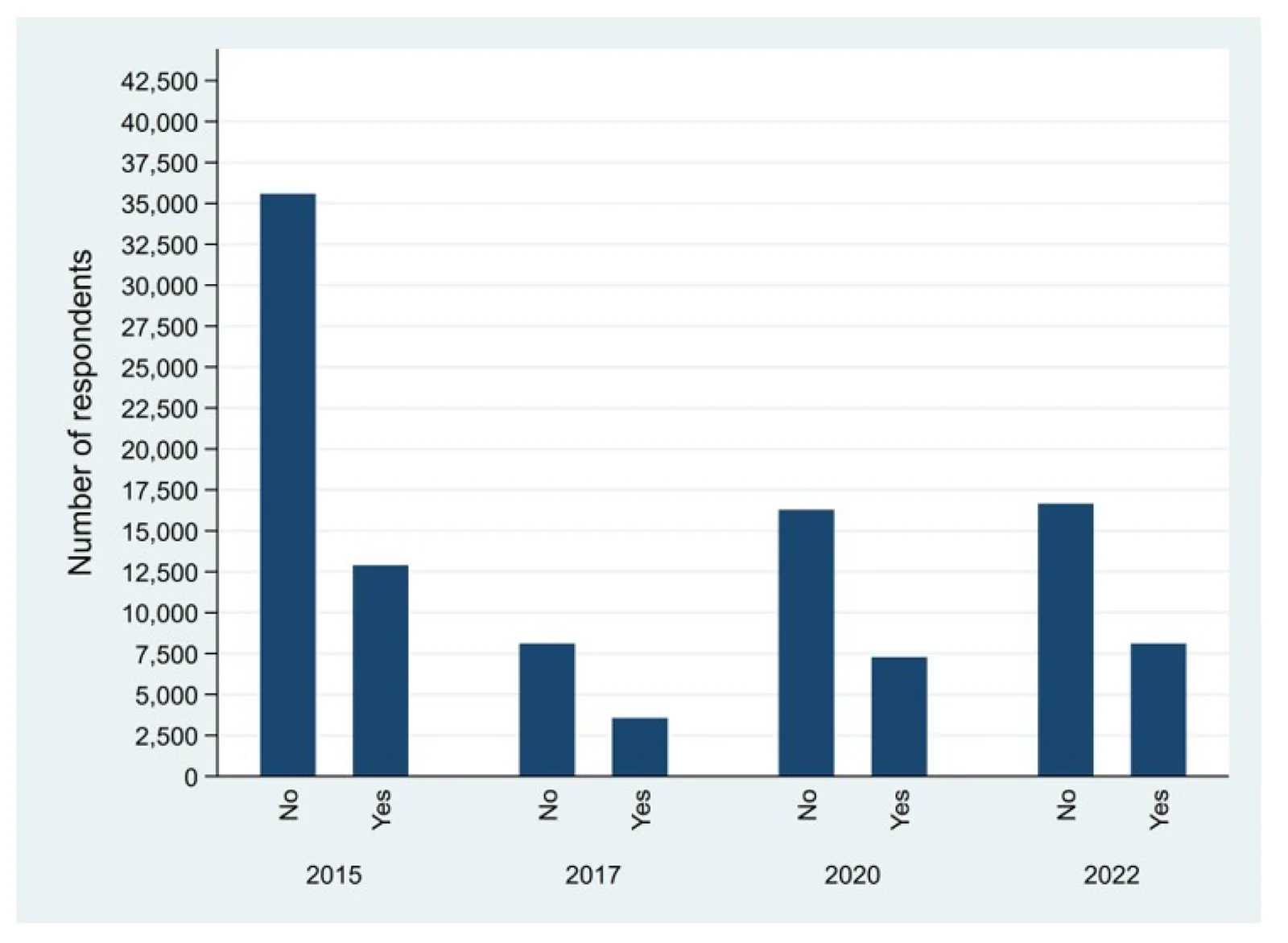
Number of Respondents per Relationship status (single or not) and per Survey year

**Figure 3:**
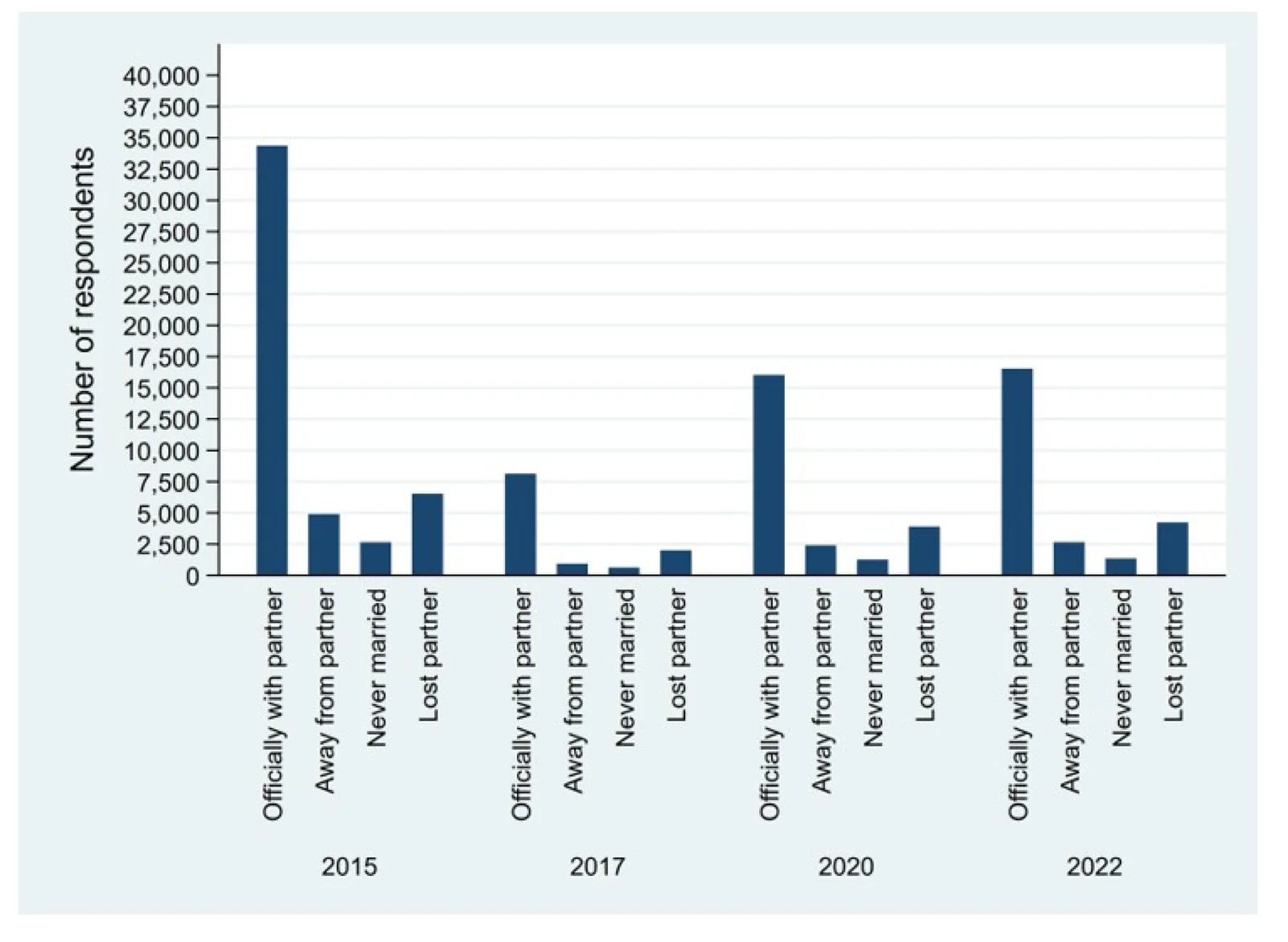
Number of Respondents per Marital status and per Survey year

**Figure 4:**
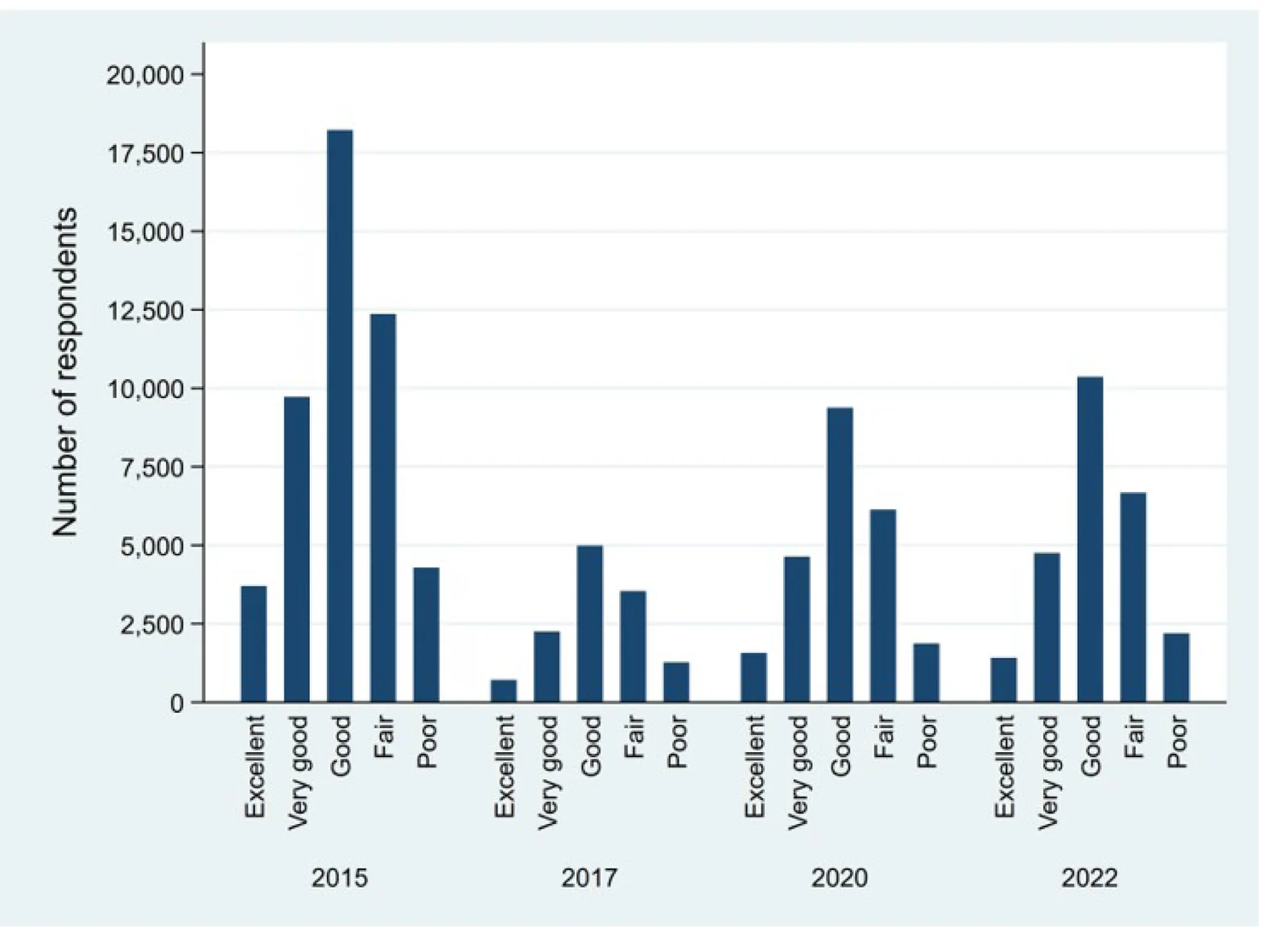
Number of Respondents per Self-perceived health status and Survey year

Figure 1 plots the number of respondents reporting physical activity and inactivity during a given survey year. The low number of respondents in 2017 is due to dropping the category Not applicable (i.e. 24,644 observations). The imbalance in the sample is acknowledged by the econometric software Stata. It is also revealed in Figs. 2 to 4.

Figure 2 plots the number of respondents reporting being single or not during a given survey year. The majority of the respondents have a relationship, implying marriage or cohabitation.

Figure 3 plots, for each survey year, the number of respondents reporting being officially with a partner, away from their partner, never married, or having lost their partner. It represents the same as Fig. 2, yet provides additional insight into the respondents who report being single.

Figure 4 plots the number of respondents reporting a given health status for a given survey year. The distribution appears roughly normal per survey year, with a majority of the respondents consistently reporting a good health status.

### Methodology

Stata 19.5 Basic Edition is used to complete the econometric analysis, which relies on logistic estimators. The regression equations vary based on two viewpoints, i.e. health and partnership. The former is covered by Limitations in activities as well as Self-perceived health status, respectively tabulated in Tables 2 and 3. Within each table, partnership is documented as Marital as well as Relationship status. Each of these four equations is regressed with a fixed- and random-effects logistic panel estimator, on top of a pooled logistic estimator. The latter is not preferred and primarily used as a benchmark for the logistic panel estimators. To explore the preference for the fixed- or random-effects estimators, Hausman and Mundlak tests are completed as a rough, yet theoretically unsupported indicator. In the previous version of this paper, the Mundlak means are integrated in the logistic panel estimators as initially documented by Gevers (2026a) to overcome the preference for the fixed-effects estimators. However, additional theoretical support for this approach is needed, thus the application is currently excluded, also in this study. Furthermore, only the pooled logistic estimators allow using calibrated longitudinal weights. These weights are preferred as they account for the demographic change in the sample. They are calculated as specified by Pacifico’s (2014) and rely on Eurostat data for mortality per year, country, age and gender. They ought to overcome issues resulting from nonresponse, refreshment as well as attrition (De Luca & Rossetti, 2018). Lastly, the estimation results reflect average effects which hold ceteris paribus.

### Literature Review

The existing literature identifies a variety of relevant findings related to physical inactivity. The latter is expected to come with a societal cost primarily arising from increased health risks. E.g., inactive Estonians are estimated to cost the public health insurance millions of euros annually. A cost that can, according to Saavaste, Baburin, Leinsalu, and Reile (2025), be minimised through prevention. Duijvestijn, de Wit, van Gils, and Wendel-Vos (2023) confirm in their systematic review the expectation that physical inactivity triggers higher healthcare costs. However, they also expect that an increased physically active population may, in the long term, result in higher healthcare costs (e.g., due to increased sports injuries). To some extent, these costs may be regarded as desirable. Li and Sun (2022) illustrate for China that physically inactive adults report 24 per cent lower odds of seeking healthcare relative to their active counterparts. They consider it primarily relevant for the early detection and treatment of diseases. Zhao et al. (2022) consider that governmental interventions ought to target the unhealthy lifestyle, in particular for those who report both inactivity and being overweight, and subsequently face higher personal out-of-pocket health expenditures and impose an additional burden on the healthcare system. Overall, overcoming the negative consequences of physical activity is not self-evident, given that the ideal healthy and healthcare-lean lifestyle is hard to define.

Beyond the financial cost, physical inactivity relates to a physical and mental cost. E.g., for the former, physical inactivity directly influences the development of chronic pain, as illustrated by Lindell and Grimby-Ekman (2022) based on a Swedish sample. The influence may be more explicit in interaction with bad sleep and stress. For the latter, physical inactivity primarily relates to depression. E.g., Nygård et al. (2025) explore physical activity among Swedes aged 65 years and older with a logistic estimator integrating gender, age, country, education, and the like. They find physical inactivity to relate for some to higher levels of psychological distress. This finding is supported by Asp, Simonsson, and Molarius (2025) who conclude that Swedish older people more often report symptoms of anxiety and self-reported depression when physically inactive. Also, Zheng, He, Xu, Zheng, and Gu (2022) consider physical inactivity strengthening depression. The influence may even hold in two directions, as concluded by Zhao et al. (2023) who conclude that a two-way causal relationship exists between physical inactivity and major depressive disorders. Evidently, besides health, a variety of other factors may explain physical inactivity. Pengpid and Peltzer (2022) find that Indian older adults are more likely to report inactivity when reporting a higher socioeconomic status, higher states of depression and/or worse health. In contrast, they consider being married, higher educated and socially active to negatively relate to physical inactivity. Primarily, the influence of marriage is of interest in this study as it is expected to come with several positive influences and benefits. E.g., Gevers (2026b) highlights the positive influence of partnership on financial distress. Stimpson et al. (2012) find that marriage results in less inactivity, in particular for those in poor health. On top, they consider inactivity determined by the extent to which resources are shared through marriage, even in a more explicit way than through cohabitation. Similarly, Hilz and Wagner (2018) consider inactive behaviour to be tempered by marriage, yet acknowledge that it may just as well trigger being overweight. The benefits of partnership also apply to Japan, where singles are more likely to be physically inactive. The latter also holds for non-working respondents. Interestingly, the study of Sumimoto et al. (2021) documents no significant effects of educational attainment on physical inactivity. In conclusion, the existing literature supports the broad expectation that health, partnership, and wealth influence physical inactivity. This expectation is the underpinning of this study for the econometric analysis completed by means of a large dataset. The latter is specified in the subsequent section.

### Data

This study uses data from SHARE, which is a project of the European Research Infrastructure Consortium (ERIC) (Bergmann et al., 2019; Börsch-Supan et al., 2013). It realises a recurring health survey focused on the effects of health, social, economic, and environmental policies. It provides information on up to 42,379 respondents from the SHARE Waves 6 to 9 (SHARE-ERIC, 2024a, 2024b, 2024c, 2024d). The respondents are aged 50 to 89 years old in 2015, and are carried forward to 2022. Table 1 on the following page summarises the variables in the sample.

The sample consists of 17 variables of which two cover the survey years, 2015, 2017, 2020, and 2022, as well as the unique identification number of each respondent. Both variables (not listed in Table 1) are preferably used as levels for the panel estimators. The remaining 15 variables are listed in Table 1. Physical inactivity is the binary dependent variable with 0 No and 1 Yes. It originally covered a category Not applicable, which has been dropped (i.e. 24,644 observations). Of the 14 independent variables, four variables are considered continuous, namely Age, Years of education, Body mass index, and Depression. A respondent is between 50 and 96 years old during the observed period, and 70 years old on average. The same respondent received 11 years of education on average, while a maximum of 30 years is observed in the sample. Overweight appears to thrive among the respondents as the average Body mass index is 27. Also underweight as well as obesity occur in the sample given, respectively, the minimum of 13 and the maximum of 99. The last continuous variable is Depression, of which its 13 categories are assumed to be on a continuous scale. The remaining 10 independent variables are used as categorical variables. Gender reports 1 Male and 2 Female. Total household income is winsorized at the 99^th^ percentile, and subsequently converted into a categorical variable with 0 No income, 1 Below country average income, and 2 Above country average income. Job situation consists of six categories, i.e. 0 Retired, 1 Employed or self-employed, 2 Unemployed, 3 Permanently sick, 4 Homemaker, and 5 Other (e.g., student or volunteer). It originally included a category Not applicable, which has been dropped (i.e. 1,089 observations). Besides the continuous health indicators Body mass index and Depression, two categoricals additionally reflect health and may be included in the econometric analysis separately. As such, the analysis integrates either the Self-perceived health status with five categories from 1 Excellent to 5 Poor, or the binary Limitation in activities with 0 Not limited or 1 Limited. A last but one categorical variable is, like the survey years, time-invariant and represents the respondent’s country of citizenship. A total of 12 European countries are covered by this variable, being Austria, Germany, Sweden, Spain, Italy, France, Denmark, Greece, Switzerland, Belgium, Czech Republic, and Poland (the original SHARE-number is referred to in Table 1). The last variables reflect relational features and are constructed as illustrated by Gevers (2026b). A first categorical is labelled Marital status and holds four categories, namely 0 Officially with partner, 1 Away from partner, 2 Never married and 3 Lost partner. The latter two are original as provided by SHARE. The former is a combination of the original SHARE-categories Married, living with spouse and Registered partnership. The category Away from partner is a combination of the original categories Married, not living with spouse and Divorced. A second relational feature is the year-on-year change in the Marital status and is documented by the variable Change in marital status, which indicates the status change relative to the previous survey year. The marital status is assumed to be unchanged in the first survey year. The variable aims to capture whether a respondent is married or not in a following survey year. The variable allows a respondent to enter and exit a marriage multiple times across the time horizon. The difference between the marriage exit and entry is of interest, implying that the variable is not interpreted relative to its (meaningless) baseline. Three categories are reported by the variable Change in marital status, i.e. 0 No longer officially together, 1 All other situations, and 2 Newly officially together. The Relationship variables are constructed in the same way as the Marital variables. However, Relationship status covers merely two categories, i.e. 0 Not single or 1 Single, and its change is covered with 0 Hooked up, 1 Unchanged, and 2 Back single. Both status, Marital as well as Relationship, reflect the same concept as supported by their strong Spearman correlation of 0.855 (see Table 4 in Appendix). As Marital status is assumed to cover the influence of marriage, and Relationship status the influence of cohabitation, both are used in the analysis, yet in separate equations. Similarly, the Spearman correlation of Limited in activities and Self-perceived health status of 0.541 is considered too high given that both variables express similar concepts. As such, also these two variables are not jointly regressed. The high Spearman correlation between Age and Job situation of −0.477 is tolerated given that it remains under a 0.600 threshold and regards different concepts and variable types.

To provide additional insight into the sample, four figures are subsequently plotted to document the primary variables of interest in this study, namely the physical inactivity, relationship status, marital status, and self-perceived health status.

Figure 1 plots the number of respondents reporting physical activity and inactivity during a given survey year. The low number of respondents in 2017 is due to dropping the category Not applicable (i.e. 24,644 observations). The imbalance in the sample is acknowledged by the econometric software Stata. It is also revealed in Figures 2 to 4.

Figure 2 plots the number of respondents reporting being single or not during a given survey year. The majority of the respondents have a relationship, implying marriage or cohabitation.

Figure 3 plots, for each survey year, the number of respondents reporting being officially with a partner, away from their partner, never married, or having lost their partner. It represents the same as Figure 2, yet provides additional insight into the respondents who report being single.

Figure 4 plots the number of respondents reporting a given health status for a given survey year. The distribution appears roughly normal per survey year, with a majority of the respondents consistently reporting a good health status.

### Methodology

Stata 19.5 Basic Edition is used to complete the econometric analysis, which relies on logistic estimators. The regression equations vary based on two viewpoints, i.e. health and partnership. The former is covered by Limitations in activities as well as Self-perceived health status, respectively tabulated in Tables 2 and 3. Within each table, partnership is documented as Marital as well as Relationship status. Each of these four equations is regressed with a fixed- and random-effects logistic panel estimator, on top of a pooled logistic estimator. The latter is not preferred and primarily used as a benchmark for the logistic panel estimators. To explore the preference for the fixed- or random-effects estimators, Hausman and Mundlak tests are completed as a rough, yet theoretically unsupported indicator. In the previous version of this paper, the Mundlak means are integrated in the logistic panel estimators as initially documented by Gevers (2026a) to overcome the preference for the fixed-effects estimators. However, additional theoretical support for this approach is needed, thus the application is currently excluded, also in this study. Furthermore, only the pooled logistic estimators allow using calibrated longitudinal weights. These weights are preferred as they account for the demographic change in the sample. They are calculated as specified by Pacifico’s (2014) and rely on Eurostat data for mortality per year, country, age and gender. They ought to overcome issues resulting from nonresponse, refreshment as well as attrition (De Luca & Rossetti, 2018). Lastly, the estimation results reflect average effects which hold ceteris paribus.

## Results

The output of the econometric analysis is documented in Tables 2 and 3. The estimates of fixed- and random-effects panel logistic estimators as well as of pooled logistic estimators are reported by means of odds ratios. The two tables solely differ in the integration of a variable explaining health. As such, Table 2 integrates Limitations in activities, and Table 3 Self-perceived health status. For robustness, merely the direction of the odds is interpreted across estimators.

Table 2 reveals the results for the logistic estimators explaining physical inactivity with personal, health and partnership characteristics. It includes Limitations in activities to additionally reflect health. A first personal characteristic, i.e. Age, reports, as expected, higher odds of being physically inactive when getting older. In turn, more Years of education report lower odds, suggesting that education may facilitate a certain awareness of the advantages of physical activity. Gender appears not to be relevant for physical activity. The relevance of income is supported by the random-effects panel and pooled logistic estimators, which suggest lower odds of reporting physical inactivity compared to those without income. Those with an income above the country average appear to less likely be physical inactive than their below country average counterparts. Yet, the fixed-effects estimators do not confirm the significance of this influence, implying that the effect is not sufficiently robust. In contrast, all categories of Job situation can be regarded as significant. Soundly, the Permanently sick are most likely physically inactive, while Homemakers and Retired respondents are least likely to be inactive. The Unemployed and Others (like volunteers or students) are expected to be inactive, yet less likely than the Permanently sick. Surprisingly, the (self-)Employed report higher odds of being physically inactive compared to the Retired ones. However, the question underlying this variable inquires about engagement in physical activity, like sports, heavy housework, or a job with physical labour. Given the inclusion of the latter activity, the higher inactivity among the employed most likely arises from the fact that physical labour is limited during their employment activities, which may cover a vast part of their activities overall. On top of the personal characteristics, three health characteristics are included. First, Limited in activities reflects whether the respondent incurred limitations because of health issues in activities during the past six months. It comes with higher odds, similar to the Permanently sick, to report physical inactivity. Surprisingly, Body mass index has no explicit influence on physical inactivity, given that its higher likelihood to report physical inactivity is not confirmed by the fixed-effects estimators. However, Depression appears significant across estimators and triggers a slight increase in the odds of reporting inactivity. Moreover, it appears to interact with the variables reflecting partnership. This effect is revealed by Marital status, as those officially without a partner relate to higher odds of reporting physical inactivity. Once more, this effect is merely confirmed by the random-effects panel and pooled logistic estimator, not their fixed-effects counterpart. Furthermore, neither the year-on-year Change in marital status nor the interaction of Change in marital status and Depression report significant odds. The alternative to Marital status is the integration of Relationship status, i.e. being single or not. This variable is regarded relevant for physical inactivity yet not by the fixed-effects estimator. Nevertheless, its year-on-year change appears to capture some of the dependent’s variance. Relative to those with an unchanged Relationship status, the respondents who hook up report significantly lower odds of being physically inactive ((4) Odds ratio 0.458, p<.10). A sound explanation is that the Change in relationship status triggers a more active social life accompanied by more physical activities. Interestingly, the Change in relationship status interacts with Depression. Being back single in combination with increasing depression appears to trigger a slight decrease in odds of reporting physical inactivity ((4) Odds ratio 0.945, p<.10; (5) Odds ratio 0.952, p<.10). A potential explanation is that singles rely on physical activity to tackle depressive symptoms. Note that the main effect of Back single is not significant. Lastly, the two time-invariant variables are tabulated. The Country of citizenship suggests that Italians are very likely to be physically inactive, while the Swedes, Danes, and Swiss mark the lowest odds of reporting physical inactivity. For the remaining, older people of Poland and Spain tend to report, like Italy, higher odds, while those of Austria, France, Greece, Belgium, and Czech Republic remain in the mid-segment. The Survey years are also documented in Table 2 and regarded being informative given that the logistic non-panel estimator ought to be able to pick up the explanatory power of this time-invariant variable. As such, solely the year 2017 suggests a significantly lower odds of reporting physical inactivity relative to the baseline 2015. An intuitive explanation may be that 2017, relative to 2015 and 2020, provided more stability and security, i.e. economic growth and no pandemic, which implied more opportunities for physical activities. To conclude, the completed tests to support the potential of the random effects estimators are referred to. Estimators (1) and (2), as well as (4) and (5), being, respectively, fixed- and random-effects panel logistic estimators, are estimated without interaction effects to perform the Hausman (1978) tests. The estimators including the Marital status and those including the Relationship status report that the coefficients of the fixed- and random-effects estimators are systematically different (respectively Chi squared 1113.014, p<0.00 and Chi squared 1117.616, p<0.00). Additionally, the Mundlak tests imply integrating the unit-specific time averages and for estimators (2) and (5) they appear to be jointly significant (respectively, Chi squared 966.58, p<0.000 and 950.32, p<0.000). As such, all tests provide a rough indication that the fixed-effects estimators are preferred unless the Mundlak correction may be applied to the random-effects logistic panel estimator. Given that additional theoretical support for the tests as well as the latter correction is needed, the result of the random-effects estimators are, for now, precautionary benchmarked against the fixed-effects logistic panel and pooled logistic estimators.

Table 3 provides the same conclusions as Table 2 given that both merely differ in a single variable, i.e. Limited in activities and Self-perceived health status. Evidently, the effects of the latter two are varying, yet also the odds ratios of several other variables lead to different conclusions. As such, the effect of Gender is rated as significant by the random-effects logistic panel estimator, implying that being Female may result in slightly higher odds of reporting physical inactivity. The effect may be relevant, yet an explanation for the higher odds is not at hand. Furthermore, the Job situation Homemaker as well as Marital status Away from partner no longer reach the ten per cent significance level. Similarly, the effects of Relationship status have strongly weakened. Merely the higher odds of reporting physical inactivity as the main effect of being single remains. The effect of Change in relationship status as well as its interaction with Depression appears to be absorbed by the more nuanced health indicator Self-perceived health status, which replaces Limitations in activities from Table 2. As such, the finding that being back single while being depressed may positively influence physical inactivity as concluded from Table 2, is not confirmed by the results in Table 3. For the time-invariant variables, an estimation difference arises from the variable Country. Table 3 suggests that Belgium, not Poland (as documented in Table 2) tends to relate to higher odds of reporting physical inactivity. As such, Poland is rather categorised in the mid-segment according to the results in Table 3. Lastly, the significant impact of Limited in activities is backed by its substitute, i.e. the Self-perceived health status. Like having limitations, being in fair or poor health status relates to higher odds of reporting physical inactivity. To conclude, like Table 2, Table 3 is accompanied by additional tests. Estimators (1) and (2), as well as (4) and (5), being the estimators including the Marital status or the Relationship status, report that the coefficients of the fixed- and random-effects estimators are systematically different (respectively Chi squared 1180.099, p<0.00 and Chi squared 1179.393, p<0.00). Also, the Mundlak tests rate significant unit-specific time averages when integrated in estimators (2) and (5). Both the Hausman and Mundlak tests suggest that the random-effects estimators are not preferred, yet applying the tests on logistic panel estimators requires further theoretical support. As such, precautionary, the results of the random-effects estimators are interpreted in comparison to the fixed-effects logistic panel and pooled logistic estimators.

## Discussion

This study reveals that partnership may interact with depression and subsequently be relevant in the context of CST. As such, the effect can be regarded as an environmental factor, which influences self-efficacy as well as its underlying self-motivation and self-regulation. The latter is explicitly a suggestion, not a finding. Regarding the findings in this study, two limitations must be referred to. Firstly, the used econometric approach resembles the approach used by Gevers (2026b) to explore financial distress. This implies that applying the Mundlak correction to safeguard the suitability of the random-effects estimator is desirable. Yet, to apply the reasoning of Mundlak on a logistic panel estimator, further theoretical support is required. The work of Wooldridge (2010, pp. 610–625) may be a good starting point. For now, the shortcoming arising from using the random-effects logistic panel estimator is compensated for in this study by solely interpreting the direction of the odds across several estimators. Secondly, measuring physical inactivity by means of a self-rated yes-no question is not preferred. Ayvat, Doğan, and Ayvat (2025) explicitly state that a stand-alone measure does not suffice, and that a “comprehensive physical inactivity assessment” is more desirable, e.g., by means of their activity scale for discriminating physical activity. Despite these limitations, the findings in this study highlight relevant drivers of physical inactivity as well as focus points for future research.

## Conclusion

Powered by three logistic estimators, this study supports a positive influence of education and partnership on physical inactivity. Ageing, illness, unemployment, limitations, and depression appear to increase the odds of reporting physical inactivity. In particular, the employed older people report increased odds relative to the retired, implying that physical labour is limited among the working older people. Additionally, physical activity as an economic benefit of marriage is suggested by this study, just as the positive influence of partnership. This may occur in the short term, given that people who hook up may less likely report physical inactivity, e.g. thanks to an augmented social life. The effect of the alternative state, being back single, in combination with increased depression, suggests a decreased likelihood of physical inactivity. Potentially, physical activity is a first resort for newly single older people to battle depression. However, these effects of marital and relationship status are not truly robust, as they appear to weaken when the self-perceived health status instead of limitation indicator is accounted for. Lastly, Swedes, Danes, and Swiss may be expected to be the least physically inactive, while Italians most likely report physical inactivity. Overall, this study reveals the importance of health and partnership for physical inactivity.

## Supplementary Material

Supplementary Files

This is a list of supplementary files associated with this preprint. Click to download.

• Appendix.docx

## Figures and Tables

**Table 1:** Descriptives

VARIABLES	Unique	Mean	Minimum	Maximum
Physical inactivity	2	-	0	1
Age	47	70.092	50.000	96.000
Years of education	30	11.021	0.000	30.000
Gender	2	-	1	2
Total household income	3	-	0	2
Job situation	6	-	1	6
Body mass index	3634	26.835	12.869	98.619
Depression	13	-	0	12
Limited in activities	2	-	0	1
Self-perceived health status	5	-	1	5
Marital status	4	-	0	3
Relationship status	2	-	0	1
Change in marital status	3	-	0	2
Change in relationship status	3	-	0	2
Country	12	-	11	28

Number of observations: 110,045

**Table 2:** Logistic estimators explaining Physical inactivity with Personal, Health (incl. Limitations in activities) and Partnership characteristics

VARIABLES	(1)	(2)	(3)	(4)	(5)	(6)
Age	1.192***	1.100***	1.060***	1.194***	1.099***	1.058***
	(0.007)	(0.003)	(0.003)	(0.007)	(0.003)	(0.003)
Years of education	0.960**	0.972***	0.978***	0.961**	0.972***	0.978***
	(0.019)	(0.004)	(0.005)	(0.019)	(0.004)	(0.005)
Male (base)						
Female	-	1.018	0.988	-	1.004	0.971
		(0.037)	(0.045)		(0.036)	(0.044)
No income (base)						
Below country average income	1.201	0.635***	0.537***	1.199	0.633***	0.536***
	(0.151)	(0.067)	(0.080)	(0.151)	(0.067)	(0.079)
Above country average income	1.150	0.519***	0.438***	1.145	0.520***	0.442***
	(0.153)	(0.056)	(0.066)	(0.153)	(0.056)	(0.067)
Retired (base)						
Employed or self-employed	1.687***	1.249***	1.016	1.699***	1.250***	1.015
	(0.173)	(0.077)	(0.085)	(0.174)	(0.077)	(0.085)
Unemployed	1.734***	1.799***	1.742***	1.745***	1.811***	1.751***
	(0.303)	(0.221)	(0.278)	(0.305)	(0.222)	(0.277)
Permanently sick	2.218***	5.423***	3.574***	2.217***	5.467***	3.583***
	(0.272)	(0.432)	(0.370)	(0.272)	(0.435)	(0.368)
Homemaker	1.177*	1.208***	1.154**	1.177*	1.203***	1.148**
	(0.115)	(0.066)	(0.078)	(0.115)	(0.066)	(0.077)
Other	1.712***	1.950***	1.425***	1.720***	1.936***	1.398***
	(0.240)	(0.194)	(0.159)	(0.241)	(0.192)	(0.156)
Not limited in activities (base)						
Limited in activities	1.998***	4.125***	2.902***	1.996***	4.130***	2.911***
	(0.099)	(0.141)	(0.126)	(0.099)	(0.141)	(0.126)
Body mass index	0.994	1.049***	1.038***	0.994	1.049***	1.038***
	(0.007)	(0.004)	(0.005)	(0.007)	(0.004)	(0.005)
Depression	1.114***	1.214***	1.145***	1.114***	1.215***	1.146***
	(0.010)	(0.008)	(0.009)	(0.010)	(0.008)	(0.009)
Officially with partner (base)						
Away from partner	0.848	1.106*	1.263***	-	-	-
	(0.290)	(0.067)	(0.109)			
Never married	0.783	1.391***	1.213**	-	-	-
	(0.439)	(0.100)	(0.107)			
Lost partner	1.038	1.094**	1.109*	-	-	-
	(0.168)	(0.049)	(0.059)			
All other situations (base)						
No longer officially together	1.306	0.822	1.048	-	-	-
	(0.314)	(0.141)	(0.247)			
Newly officially together	0.643	1.649	1.348	-	-	-
	(0.352)	(0.643)	(0.558)			
No longer officially together#Depression	0.938	0.979	0.969	-	-	-
	(0.037)	(0.032)	(0.037)			
Newly officially together#Depression	1.055	0.966	0.968	-	-	-
	(0.127)	(0.099)	(0.090)			
Not single (base)						
Single	-	-	-	1.027	1.141***	1.196***
				(0.145)	(0.044)	(0.059)
Unchanged (base)						
Hooked up	-	-	-	0.458*	0.535	0.603
				(0.215)	(0.235)	(0.292)
Back single	-	-	-	1.131	0.948	0.980
				(0.228)	(0.137)	(0.189)
Hooked up#Depression	-	-	-	1.198	1.143	0.967
				(0.137)	(0.106)	(0.102)
Back single#Depression	-	-	-	0.945*	0.952*	0.959
				(0.031)	(0.027)	(0.033)
Austria (base)						
Germany	-	0.793***	0.944	-	0.791***	0.942
		(0.070)	(0.080)		(0.070)	(0.080)
Sweden	-	0.383***	0.487***	-	0.383***	0.488***
		(0.037)	(0.050)		(0.038)	(0.050)
Spain	-	2.422***	2.194***	-	2.416***	2.167***
		(0.194)	(0.201)		(0.193)	(0.198)
Italy	-	6.268***	4.283***	-	6.241***	4.222***
		(0.493)	(0.323)		(0.490)	(0.316)
France	-	1.404***	1.318***	-	1.403***	1.312***
		(0.121)	(0.105)		(0.121)	(0.105)
Denmark	-	0.647***	0.782***	-	0.648***	0.784***
		(0.063)	(0.072)		(0.064)	(0.073)
Greece	-	1.260***	1.298***	-	1.250***	1.280***
		(0.106)	(0.101)		(0.105)	(0.100)
Switzerland	-	0.449***	0.596***	-	0.450***	0.598***
		(0.049)	(0.062)		(0.049)	(0.062)
Belgium	-	1.504***	1.315***	-	1.499***	1.318***
		(0.122)	(0.103)		(0.121)	(0.104)
Czech Republic	-	1.308***	1.193*	-	1.293***	1.183
		(0.109)	(0.124)		(0.107)	(0.123)
Poland	-	2.291***	1.793***	-	2.262***	1.752***
		(0.219)	(0.158)		(0.216)	(0.153)
2015 (base)						
2017	-	-	0.797***	-	-	0.806***
			(0.036)			(0.036)
2020	-	-	0.964	-	-	0.978
			(0.046)			(0.046)
2022	-	-	0.991	-	-	1.008
			(0.043)			(0.044)

Number of observations	17,262	110,045	110,007	17,262	110,045	110,007
Number of respondents	5,962	48,341	-	5,962	48,341	-
Calibrated longitudinal weights	No	No	Yes	No	No	Yes
Panel structure	Yes	Yes	No	Yes	Yes	No
Effects	Fixed	Random	-	Fixed	Random	-
Non-zero panel level variance	-	Yes	-	-	Yes	-
Errors in parentheses	Standard	Robust	Cluster	Standard	Robust	Cluster

The estimation results are provided by logistic estimators which report odds ratios. Physical inactivity is the binary dependent variable with 0 No and 1 Yes. Cluster indicates standard errors which account for the within respondent correlation. The Panel structure consists of the respondent’s unique identifier and the survey year. The estimators use SHARE-data from Imputation 1. The fixed-effects panel estimators in columns 2 and 5 drop 42,379 respondents (i.e. 92,783 observations) as they consistently report the same value for the dependent variable over time. A hashtag indicates an interaction effect.

*** p<0.01,

** p<0.05,

* p<0.10

**Table 3:** Logistic estimators explaining Physical inactivity with Personal, Health (incl. Self-perceived health status) and Partnership characteristics

VARIABLES	(1)	(2)	(3)	(4)	(5)	(6)
Age	1.188***	1.097***	1.059***	1.190***	1.096***	1.058***
	(0.007)	(0.003)	(0.003)	(0.007)	(0.003)	(0.003)
Years of education	0.959**	0.982***	0.984***	0.959**	0.982***	0.984***
	(0.019)	(0.004)	(0.006)	(0.019)	(0.004)	(0.006)
Male (base)						
Female	-	1.137***	1.052	-	1.123***	1.034
		(0.040)	(0.049)		(0.040)	(0.048)
No income (base)						
Below country average income	1.163	0.565***	0.478***	1.160	0.565***	0.478***
	(0.147)	(0.058)	(0.074)	(0.147)	(0.058)	(0.073)
Above country average income	1.111	0.486***	0.401***	1.105	0.489***	0.408***
	(0.149)	(0.051)	(0.063)	(0.149)	(0.052)	(0.063)
Retired (base)						
Employed or self-employed	1.660***	1.348***	1.114	1.671***	1.349***	1.110
	(0.171)	(0.083)	(0.094)	(0.172)	(0.083)	(0.094)
Unemployed	1.739***	1.834***	1.796***	1.746***	1.843***	1.801***
	(0.307)	(0.222)	(0.305)	(0.308)	(0.223)	(0.303)
Permanently sick	2.138***	4.206***	3.064***	2.139***	4.234***	3.065***
	(0.269)	(0.335)	(0.340)	(0.269)	(0.338)	(0.338)
Homemaker	1.142	1.217***	1.180**	1.139	1.214***	1.178**
	(0.113)	(0.067)	(0.082)	(0.112)	(0.066)	(0.082)
Other	1.604***	1.858***	1.452***	1.607***	1.849***	1.427***
	(0.227)	(0.187)	(0.172)	(0.228)	(0.186)	(0.168)
Excellent self-perceived health (base)						
Very Good	0.816	0.963	1.352**	0.820	0.964	1.345**
	(0.117)	(0.094)	(0.194)	(0.118)	(0.094)	(0.193)
Good	0.971	1.418***	1.477***	0.972	1.420***	1.474***
	(0.135)	(0.130)	(0.193)	(0.135)	(0.130)	(0.193)
Fair	1.554***	3.766***	3.194***	1.556***	3.773***	3.189***
	(0.220)	(0.348)	(0.418)	(0.221)	(0.349)	(0.417)
Poor	3.967***	21.203***	12.002***	3.966***	21.251***	12.009***
	(0.595)	(2.101)	(1.633)	(0.596)	(2.105)	(1.632)
Body mass index	0.997	1.050***	1.037***	0.997	1.050***	1.037***
	(0.008)	(0.004)	(0.005)	(0.008)	(0.004)	(0.005)
Depression	1.081***	1.124***	1.078***	1.081***	1.124***	1.078***
	(0.010)	(0.008)	(0.009)	(0.010)	(0.008)	(0.009)
Officially with partner (base)						
Away from partner	0.904	1.070	1.213**	-	-	-
	(0.310)	(0.064)	(0.108)			
Never married	0.850	1.368***	1.228**	-	-	-
	(0.500)	(0.097)	(0.110)			
Lost partner	1.053	1.117**	1.124**	-	-	-
	(0.172)	(0.050)	(0.063)			
All other situations (base)						
No longer officially together	1.224	0.865	1.097	-	-	-
	(0.301)	(0.149)	(0.263)			
Newly officially together	0.676	1.880	1.645	-	-	-
	(0.381)	(0.729)	(0.696)			
No longer officially together#Depression	0.948	0.979	0.970	-	-	-
	(0.038)	(0.032)	(0.039)			
Newly officially together#Depression	1.087	0.974	0.968	-	-	-
	(0.135)	(0.097)	(0.093)			
Not single (base)						
Single	-	-	-	1.022	1.147***	1.208***
				(0.146)	(0.044)	(0.062)
Unchanged (base)						
Hooked up	-	-	-	0.467	0.596	0.572
				(0.221)	(0.266)	(0.264)
Back single	-	-	-	1.052	0.924	0.925
				(0.216)	(0.134)	(0.181)
Hooked up#Depression	-	-	-	1.182	1.120	0.984
				(0.135)	(0.107)	(0.088)
Back single#Depression	-	-	-	0.962	0.968	0.973
				(0.032)	(0.028)	(0.034)
Austria (base)						
Germany	-	0.714***	0.851*	-	0.714***	0.850*
		(0.062)	(0.074)		(0.062)	(0.074)
Sweden	-	0.404***	0.487***	-	0.404***	0.488***
		(0.039)	(0.051)		(0.039)	(0.051)
Spain	-	1.815***	1.756***	-	1.816***	1.740***
		(0.141)	(0.165)		(0.141)	(0.164)
Italy	-	5.288***	3.912***	-	5.283***	3.873***
		(0.403)	(0.300)		(0.402)	(0.295)
France	-	1.267***	1.213**	-	1.270***	1.210**
		(0.108)	(0.099)		(0.108)	(0.099)
Denmark	-	0.674***	0.776***	-	0.678***	0.780***
		(0.065)	(0.073)		(0.066)	(0.073)
Greece	-	0.950	1.027	-	0.945	1.015
		(0.079)	(0.082)		(0.078)	(0.081)
Switzerland	-	0.509***	0.626***	-	0.510***	0.627***
		(0.054)	(0.067)		(0.054)	(0.067)
Belgium	-	1.760***	1.495***	-	1.759***	1.501***
		(0.139)	(0.120)		(0.139)	(0.120)
Czech Republic	-	1.184**	1.122	-	1.173**	1.116
		(0.096)	(0.119)		(0.095)	(0.118)
Poland	-	1.617***	1.423***	-	1.606***	1.397***
		(0.153)	(0.130)		(0.152)	(0.126)
2015 (base)						
2017	-	-	0.801***	-	-	0.812***
			(0.038)			(0.038)
2020	-	-	1.029	-	-	1.046
			(0.050)			(0.051)
2022	-	-	1.058	-	-	1.077
			(0.048)			(0.049)

Number of observations	17,262	110,045	110,007	17,262	110,045	110,007
Number of respondents	5,962	48,341	-	5,962	48,341	-
Calibrated longitudinal weights	No	No	Yes	No	No	Yes
Panel structure	Yes	Yes	No	Yes	Yes	No
Effects	Fixed	Random	-	Fixed	Random	-
Non-zero panel level variance	-	Yes	-	-	Yes	-
Errors in parentheses	Standard	Robust	Cluster	Standard	Robust	Cluster

The estimation results are provided by logistic estimators which report odds ratios. Physical inactivity is the binary dependent variable with 0 No and 1 Yes. Cluster indicates standard errors which account for the within respondent correlation. The Panel structure consists of the respondent’s unique identifier and the survey year. The estimators use SHARE-data from Imputation 1. The fixed-effects panel estimators in columns 2 and 5 drop 42,379 respondents (i.e. 92,783 observations) as they consistently report the same value for the dependent variable over time. A hashtag indicates an interaction effect.

*** p<0.01,

** p<0.05,

* p<0.10

## Data Availability

To provide insight into the longitudinal analysis completed in this article, do- and log-files of Stata are available via https://github.com/hansgevers/physicalinactivityandmarriage.

## Appendix

**Table 4:** reveals the Spearman correlation between the continuous and categorical variables, except for the respondent’s unique identification number and the survey years.

1	VARIABLES	1	2	3	4	5	6	7	8	9	10	11	12	13	14	15
	Physical inactivity	1.000	-	-	-	-	-	-	-	-	-	-	-	-	-	-
2	Limited in activities	0.251	1.000	-	-	-	-	-	-	-	-	-	-	-	-	-
3	Self-perceived health status	0.317	0.541	1.000	-	-	-	-	-	-	-	-	-	-	-	-
4	Total household income	−0.107	−0.110	−0.164	1.000	-	-	-	-	-	-	-	-	-	-	-
5	Relationship status	0.093	0.101	0.108	−0.363	1.000	-	-	-	-	-	-	-	-	-	-
6	Marital status	0.086	0.095	0.098	−0.304	0.855	1.000	-	-	-	-	-	-	-	-	-
7	Change in marital status	−0.019	−0.022	−0.029	0.076	−0.197	−0.229	1.000	-	-	-	-	-	-	-	-
8	Change in relationship status	0.025	0.029	0.035	−0.087	0.246	0.152	−0.675	1.000	-	-	-	-	-	-	-
9	Job situation	0.003	−0.083	−0.068	−0.004	−0.048	−0.044	0.017	−0.019	1.000	-	-	-	-	-	-
10	Age	0.206	0.214	0.257	−0.191	0.209	0.199	−0.082	0.085	−0.477	1.000	-	-	-	-	-
11	Years of education	−0.157	−0.099	−0.222	0.222	−0.071	−0.074	0.031	−0.026	−0.015	−0.230	1.000	-	-	-	-
12	Gender	0.037	0.040	0.027	−0.103	0.201	0.192	−0.040	0.037	0.178	−0.027	−0.073	1.000	-	-	-
13	Country	−0.002	−0.011	0.000	−0.016	0.027	0.030	0.004	0.002	−0.019	−0.051	0.061	0.016	1.000	-	-
14	Body mass index	0.052	0.106	0.149	−0.038	−0.023	−0.017	0.009	−0.010	−0.010	−0.025	−0.101	−0.091	0.052	1.000	-
15	Depression	0.209	0.331	0.403	−0.120	0.144	0.133	−0.067	0.074	0.036	0.110	−0.113	0.182	0.012	0.027	1.000

## References

[1] Asp M, Simonsson B, Molarius A (2025) Physical Activity, Physical Mobility, and Mental Health Among Persons 70 Years or Older: Results From a Large Population-Based Study in Sweden. Archives Public Health 83(1):225. 10.1186/s13690-025-01718-w PMC12418685 40926247

[2] Averett SL, Argys LM, Sorkin J (2013) In Sickness and in Health: An Examination of Relationship Status and Health Using Data From the Canadian National Public Health Survey. Rev Econ Househ 11(4):599–633. 10.1007/s11150-012-9143-z

[3] Ayvat F, Doğan M, Ayvat E (2025) Detecting Inactivity in Aging Populations: The Discriminative Potential of the Physical Activity Scale for the Elderly. BMC Public Health 25:1–7. 10.1186/s12889-025-24388-3 40866950 PMC12382271

[4] Bandura A (1994) Social Cognitive Theory and Exercise of Control over HIV Infection. In DiClemente R. J. & Peterson J. L. (Eds.), Preventing AIDS: Theories and Methods of Behavioral Interventions (pp. 25–59). Springer US. 10.1007/978-1-4899-1193-3_3

[5] Bergmann M, Kneip T, De Luca G, Scherpenzeel A (2019) Survey Participation in the Survey of Health, Ageing and Retirement in Europe (SHARE), Wave 1–7 (based on Release 7.0.0.). In SHARE Working Paper Series: 41–2019. Munich: Max Planck Institute for Social Law and Social Policy

[6] Börsch-Supan A, Brandt M, Hunkler C, Kneip T, Korbmacher J, Malter F, Zuber S (2013) Data Resource Profile: The Survey of Health, Ageing and Retirement in Europe (SHARE). Int J Epidemiol 42(4):992–1001. 10.1093/ije/dyt088 23778574 PMC3780997

[7] De Luca G, Rossetti C (2018) Survey of Health, Ageing and Retirement in Europe (SHARE) Computing Calibrated Weights. https://share-eric.eu/fileadmin/user_upload/pdf_documentation/SHARE_calibrated_weights_user_guide.pdf

[8] Duijvestijn M, Wit GAd, van Gils PF, Wendel-Vos GCW (2023) Impact of Physical Activity on Healthcare Costs: A Systematic Review. BMC Health Serv Res 23:1–13. 10.1186/s12913-023-09556-8 37268930 PMC10239135

[9] Gevers H (2026a) Exposing the Value of Receiving and Giving Help Across 12 European Countries: A Longitudinal Analysis of Self-Perceived Health. Preprints. 10.20944/preprints202602.1956.v5

[10] Gevers H (2026b) The Impact of Marriage and Cohabitation on Financial Distress: Evidence from SHARE 2015 to 2022 for 16 European Countries. Preprints. 10.20944/preprints202606.1544.v1

[11] Hausman JA (1978) Specification Tests in Econometrics. Econometrica 46(6):1251–1271. 10.2307/1913827

[12] Hilz R, Wagner M (2018) Marital Status, Partnership and Health Behaviour: Findings from the German Ageing Survey (DEAS). Comp Popul Stud / Z für Bevölkerungswissenschaft 43:65–97. 10.12765/cpos-2018-08

[13] Li C, Sun J (2022) The Impact of Current Smoking, Regular Drinking, and Physical Inactivity on Health Care-Seeking Behavior in China. BMC Health Serv Res 22:1–8. 10.1186/s12913-022-07462-z PMC8751354 35012543

[14] Lindell M, Grimby-Ekman A (2022) Stress, Non-Restorative Sleep, and Physical Inactivity as Risk Factors for Chronic Pain in Young Adults: A Cohort Study. PLoS ONE 17(1):e0262601. 10.1371/journal.pone.0262601 PMC8782303 35061825

[15] Nygård S, Tiikkaja S, Lönnberg L, Pellas J, Tonkonogi M, Liljeroos M, Arkkukangas M (2025) Psychological Distress, Psychosocial Factors, and Physical Inactivity Among Older Women and Men in Sweden: A Population-Based Study. BMC Public Health 25:1–10. 10.1186/s12889-025-24868-6 41126176 PMC12542086

[16] Pacifico D (2014) sreweight: A Stata Command to Reweight Survey Data to External Totals. Stata J 14(1):4–21

[17] Pengpid S, Peltzer K (2022) Prevalence and Associated Factors of Physical Inactivity Among Middle-Aged and Older Adults in India: Results of a National Cross-Sectional Community Survey. BMJ Open 12(8). 10.1136/bmjopen-2021-058156 PMC9422873 36028277

[18] Pollard M, Mullan Harris K, May (2013) 24, Nonmarital Cohabitation, Marriage, and Health Among Adolescents and Young Adults. RAND Working Paper Series WR-997. https://ssrn.com/abstract=2284392 or 10.2139/ssrn.2284392

[19] Saavaste J, Baburin A, Mall L, Reile R (2025) The Healthcare Costs of Physical Inactivity Among Adults in Estonia. BMC Public Health 25:1–8. 10.1186/s12889-025-22875-1 40329258 PMC12054277

[20] SHARE-ERIC (2024a) Survey of Health, Ageing and Retirement in Europe (SHARE) Wave 6 (Version 9.0.0). 10.6103/SHARE.w6.900

[21] SHARE-ERIC (2024b) Survey of Health, Ageing and Retirement in Europe (SHARE) Wave 7 (Version 9.0.0). 10.6103/SHARE.w7.900

[22] SHARE-ERIC (2024c) Survey of Health, Ageing and Retirement in Europe (SHARE) Wave 8 (Version 9.0.0). 10.6103/SHARE.w8.900

[23] SHARE-ERIC (2024d) Survey of Health, Ageing and Retirement in Europe (SHARE) Wave 9 (Version 9.0.0). 10.6103/SHARE.w9.900

[24] Stimpson JP, Wilson FA, Peek MK (2012) Marital Status, the Economic Benefits of Marriage, and Days of Inactivity due to Poor Health. International Journal of Population Research, 2012. 10.1155/2012/568785

[25] Sumimoto Y, Yanagita M, Miyamatsu N, Okuda N, Nishi N, Nakamura Y,… N. D. R. G (2021) Association Between Socioeconomic Status and Physical Inactivity in a General Japanese Population: NIPPON DATA2010. PLoS ONE 16(7). 10.1371/journal.pone.0254706 PMC8282078 34265008

[26] Wood R, Bandura A (1989) Social Cognitive Theory Of Organizational Management. Acad Manage Acad Manage Rev 14(3):361

[27] Wooldridge JM (2010) Econometric Analysis of Cross Section and Panel Data. MIT Press

[28] Zhao G, Lu Z, Sun Y, Kang Z, Feng X, Liao Y, Yue W (2023) Dissecting the Causal Association Between Social or Physical Inactivity and Depression: a Bidirectional Two-Sample Mendelian Randomization Study. Translational Psychiatry 13(1):194. 10.1038/s41398-023-02492-5 37291091 PMC10250407

[29] Zhao Y, He L, Marthias T, Ishida M, Anindya K, Desloge A, Lee JT (2022) Out-Of-Pocket Expenditure Associated with Physical Inactivity, Excessive Weight, and Obesity in China: Quantile Regression Approach. Obes Facts 15(3):416–427. 10.1159/000522433 35249040 PMC9209956

[30] Zheng H, He Q, Xu H, Zheng X, Gu Y (2022) Lower Grip Strength and Insufficient Physical Activity Can Increase Depressive Symptoms Among Middle-Aged and Older European Adults: A Longitudinal Study. BMC Geriatr 22(1):696 35996095 10.1186/s12877-022-03392-x PMC9396791

